# Supplementary material for: Reconciling Mining with the Conservation of Cave Biodiversity: A Quantitative Baseline to Help Establish Conservation Priorities
Source: PLoS One. 2016 Dec 20;11(12):e0168348. doi: 10.1371/journal.pone.0168348 (PMC5173368; doi:10.1371/journal.pone.0168348)
Supplement: S1 Dataset — (ZIP) [file pone.0168348.s002.zip › Taxa/Serra Norte/SN_2013/N4E/Lista N4E-37.pdf]

CAVIDADE N4E-0037

Classe	Ordem	Fam/Outros	Gên/Outros	Espécie	S-U	U-S	Zon.
Arachnida	Acari	Macronyssidae		sp.1	X		P
Arachnida	Araneae	Ctenidae		jovem		1	
Arachnida	Araneae	Filistatidae		jovem	X	X	P
Arachnida	Araneae	Pholcidae	*Mesabolivar*	sp.1	X	X	
Arachnida	Araneae	Scytodidae	*Scytodes*	sp.		X	
Insecta	Coleoptera	Tenebrionidae		sp.		X	
Insecta	Coleoptera			jovem	X		P
Insecta	Diptera	Psychodidae	*Sciopemyia*	*sordellii*	X	X	
Insecta	Heteroptera	Reduviidae	*Ploiaria*	sp.		X	
Insecta	Homoptera	Cixiidae		sp.		X	
Insecta	Hymenoptera	Formicidae	*Camponotus*	*crassus*		X	
Insecta	Hymenoptera	Formicidae	*Crematogaster*	sp.1	X		P
Insecta	Hymenoptera	Formicidae	*Gnamptogenys*	*striatula*		X	
Insecta	Hymenoptera	Formicidae	*Pachycondyla*	sp.1	X		E
Insecta	Hymenoptera	Nyssonidae		sp.1	X		
Insecta	Hymenoptera	Vespidae		sp.1	X		
Insecta	Isoptera	Termitidae		sp.		X	
Insecta	Lepidoptera	Tineoidea		sp.1	X	X	P
Insecta	Neuroptera	Myrmeleontidae		jovem	2	X	E
Insecta	Orthoptera	Phalangopsidae	*Paraclodes*	sp.	1	X	E
Insecta	Orthoptera	Phalangopsidae	*Phalangopsis*	sp.		X	P
Insecta	Psocoptera	Psocomorpha		jovem		X	
Ave				sp.	1		P
Mammalia	Chiroptera	Emballonuridae	*Peropteryx*	*kappleri*	2	2	E
